# Supplementary material for: Exploring Genetics by Environment Interactions in Some Rice Genotypes across Varied Environmental Conditions
Source: Plants (Basel). 2023 Dec 26;13(1):74. doi: 10.3390/plants13010074 (PMC10780751; doi:10.3390/plants13010074)
Supplement: Supplementary file 1 [file plants-13-00074-s001.zip › plants-2730217-supplementary.pdf]

Table S1 The monthly maximum and minimum temperature (°C) as well as relative humidity (%) at four locations during 2021 and 2022 rice seasons.

Month	Kafr EL-Sheikh Governorate						Gharbya Governorate						Alexandria Governorate						New Valley Governorate					
	Air Temp. 2021		RH % 2021	Air Temp. 2022		RH % 2022	Air Temp. 2021		RH % 2021	Air Temp. 2022		RH % 2022	Air Temp. 2021		RH % 2021	Air Temp. 2022		RH % 2022	Air Temp. 2021		RH % 2021	Air Temp. 2022		RH % 2022
	Max	Min		Max	Min		Max	Min		Max	Min		Max	Min		Max	Min		Max	Min		Max	Min	
**May**	**32.5**	**24.8**	**49.55**	**30.0**	**21.8**	**53.85**	**37.1**	**17.9**	**46.4**	**34.1**	**16.8**	**51.3**	**28.30**	**19.00**	**56.47**	**26.3**	**17.96**	**61.22**	**41.1**	**23.3**	**17.04**	**37.8**	**22.3**	**20.33**
**June**	**32.1**	**25.5**	**51.54**	**33.0**	**25.7**	**53.04**	**36.9**	**19.8**	**50.1**	**37.8**	**21.1**	**50.8**	**28.88**	**20.45**	**60.39**	**27.9**	**19.35**	**62.61**	**41.9**	**24.6**	**22.44**	**40.2**	**24.7**	**23.10**
**July**	**34.7**	**28.0**	**52.94**	**33.4**	**25.9**	**53.53**	**39.7**	**22.8**	**51.0**	**38.9**	**22.1**	**51.1**	**29.02**	**25.82**	**60.77**	**28.27**	**25.05**	**61.36**	**42.4**	**25.9**	**22.08**	**40.7**	**24.1**	**22.87**
**August**	**35.6**	**28.3**	**55.11**	**34.4**	**25.9**	**55.52**	**40.4**	**23.5**	**52.8**	**38.6**	**23.2**	**54.0**	**31.65**	**26.06**	**63.02**	**30.34**	**25.77**	**61.44**	**42.1**	**25.2**	**22.70**	**41.2**	**25.5**	**25.40**
**September**	**32.5**	**25.1**	**55.99**	**33.0**	**25.9**	**55.36**	**36.3**	**21.9**	**55.4**	**37.0**	**22.2**	**54.6**	**31.40**	**25.95**	**61.19**	**32.22**	**26.00**	**60.50**	**38.2**	**24.4**	**30.88**	**39.8**	**23.9**	**27.03**

Table S2 Physical and chemical properties of experimental field soils at four locations in the rice growing seasons.

Characters	Kafr EL-Sheikh Governorate		Gharbya Governorate		Alexandria Governorate		New Valley Governorate	
	**2021**	**2022**	**2021**	**2022**	**2021**	**2022**	**2021**	**2022**
**chemical analysis**								
PH	8.00	8.30	7.98	8.12	8.10	7.98	7.25	7.50
Organic matter (%)	1.45	1.58	--	--	1.60	1.67	--	--
**Soluble Cations, meq/l.**								
Ca^{++}	7.40	7.20	2.05	2.70	8.50	9.54	1.03	0.95
Mg^{++}	2.85	2.65	1.85	2.30	10.50	9.35	1.00	1.20
Na^{+}	13.10	14.10	3.55	4.48	59.10	58.94	0.98	1.05
K^{+}	1.03	1.21	0.31	0.28	1.10	0.93	0.19	0.17
**Soluble Anions, meq/l.**								
Co_3^{--}	--	--	--	--	--	--	--	--
Hco_3^{-}	4.85	5.40	3.03	4.29	4.00	5.76	0.90	0.88
So_4^{--}	7.80	8.28	1.58	2.61	8.49	10.10	1.49	1.20
Cl^{-}	11.50	11.75	2.15	2.86	74.5	70.78	0.75	1.43
**Physical analysis**								
Sand (%)	13.28	13.73	13.61	12.11	44.20	44.15	59.32	41.98
Clay (%)	56.05	55.55	49.63	50.69	29.76	30.25	21.48	32.00
Silt (%)	32.49	30.44	28.31	30.60	26.10	26.84	10.13	10.58
$CaCo_3$	3.85	3.35	4.05	3.77	4.85	4.57	2.15	1.75
Soil texture	Clay	Clay	Clay	Clay	Sand clay loam	Sand clay loam	Loam sandy	Clay sandy

Table S3 Genotypes mean performance under the eight different environments

GEN	Kafr EL-Sheikh Governorate		Alexandria Governorate		Gharbya Governorate		New Valley Governorate		Mean
	Env1	Env2	Env3	Env4	Env5	Env6	Env7	Env8	
G1	956.13	943.21	244.88	238.47	970.20	967.98	263.33	247.08	603.91
G2	1082.49	1106.09	636.67	654.13	1164.23	1150.00	864.50	834.00	936.51
G3	1117.28	1173.48	690.40	692.50	1198.76	1209.91	766.50	746.83	949.46
G4	1080.37	1103.34	438.67	423.78	1102.34	1052.19	478.00	496.58	771.91
G5	1010.26	1026.16	349.51	354.96	1040.65	1058.25	440.68	433.92	714.30
G6	1036.95	1072.50	498.55	512.11	1122.37	1123.80	540.33	539.58	805.78
G7	1017.43	1026.65	401.47	400.60	1003.77	1025.50	420.28	410.67	713.30
G8	1008.38	1046.45	395.80	397.18	1043.09	1049.54	422.50	432.50	724.43
G9	1036.45	1039.94	485.59	487.47	1132.82	1103.17	688.25	698.08	833.97
G10	1087.34	1100.60	464.03	456.60	1172.00	1173.89	488.17	477.25	802.48
G11	1103.74	1140.28	502.64	496.65	1193.71	1154.55	512.68	527.68	828.99
G12	919.42	942.61	700.58	683.86	872.67	871.55	688.42	643.58	790.34
G13	914.32	940.11	515.37	515.59	956.26	982.87	677.25	681.58	772.92
G14	821.68	841.54	423.53	414.99	889.79	893.23	585.75	583.75	681.78
G15	1035.00	1052.96	690.68	677.47	1023.89	1028.85	905.67	893.75	913.53
G16	905.47	903.30	511.88	510.64	999.79	981.91	629.33	615.68	757.25
G17	806.39	829.56	401.91	407.13	858.99	864.18	485.83	503.85	644.73
G18	1001.77	1002.62	776.58	779.73	989.08	994.21	717.31	710.89	871.52
G19	801.32	803.29	595.87	597.04	873.99	878.17	742.25	767.93	757.48
G20	800.93	800.49	459.81	469.33	844.84	866.52	606.17	593.83	680.24
G21	977.03	987.55	600.93	600.04	955.20	947.51	922.33	899.15	861.22
G22	1005.99	1057.55	606.55	592.30	1062.83	1091.11	802.42	783.27	875.25
G23	814.95	815.60	537.14	553.35	883.60	870.86	646.43	621.94	717.98
G24	1008.46	1027.03	583.62	585.37	1069.51	1070.58	754.67	753.00	856.53
G25	908.32	914.34	560.36	558.69	924.56	930.94	637.83	659.54	761.82
G26	801.91	814.18	500.42	500.08	793.66	851.56	647.05	659.00	695.98
G27	1017.16	1031.17	601.76	595.53	1043.27	1078.26	758.58	730.00	856.97
G28	1015.52	1020.59	585.40	580.56	1034.80	1024.20	729.75	767.08	844.74
G29	1016.34	1044.40	484.09	489.32	966.85	978.82	532.83	548.17	757.60
G30	796.15	804.46	598.68	600.40	860.13	873.80	748.37	753.08	754.38
G31	735.50	745.76	494.01	493.27	770.16	733.51	592.92	651.67	652.10
G32	808.17	817.21	295.46	298.43	776.90	791.47	327.67	326.68	555.25
G33	893.87	896.98	594.84	597.45	965.19	966.85	769.33	777.92	807.80
G34	826.65	821.27	653.17	653.38	872.50	894.20	765.09	750.92	779.65

Table S4 Genotypes and environments overall mean performance and their PC1.

**Entry**	Name	**PC1**
G1	Giza 177	-12.5
G2	Giza 178	0.9
G3	Giza 179	-2.6
G4	Sakha 101	-9.1
G5	Sakha 102	-9.4
G6	Sakha 104	-7.0
G7	Sakha 105	-8.8
G8	Sakha 106	-9.2
G9	Sakha 107	-3.1
G10	Sakha 108	-10.6
G11	Sakha Super 300	-9.8
G12	IRAT 170	5.4
G13	A22	1.6
G14	Nerica 9	1.3
G15	IET 1444	5.9
G16	Nerica 7	0.3
G17	Moroberekan	-0.2
G18	GZ 1368	4.0
G19	Azucena	8.5
G20	IRAT 112	3.5
G21	N22	7.6
G22	IR65600-77	1.5
G23	IR69116	4.7
G24	IR12G3213	0.8
G25	IR69432	2.5
G26	IR6500-127	5.7
G27	IR11L236	0.8
G28	IR12G3222	1.4
G29	Sakha 109	-4.1
G30	Vandana	12.0
G31	Dular	7.4
G32	Sakha 103	-4.1
G33	Giza 181	5.6
G34	Giza 182	9.0
E1	Kafrelsheikh-2021	-13.0
E2	Kafrelsheikh-2022	-13.7
E3	Alexandria-2021	8.8
E4	Alexandria-2022	8.9
E5	Gharbya-2021	-12.9
E6	Gharbya-2022	-12.2
E7	New Valley-2021	16.9
E8	New Valley-2022	17.1

Table S5 Genotypes rankings based on yield performance and several stability indices.

GEN	Y_R	Shukla_R	Wi_g_R	Wi_f_R	Wi_u_R	Ecoval_R	Sij_R	Pi_a_R	Pi_f_R	Pi_u_R	Gai_R	S1_R	S2_R	S3_R	S6_R	N1_R
**Giza 177**	33	34	34	17	34	34	33	33	19	34	34	4	34	31	34	34
**Giza 178**	2	10	2	3	3	10	18	1	4	2	2	33	9	1	1	7.5
**Giza 179**	1	9	1	1	4	9	9	2	1	3	1	4	10.5	8	4	11
**Sakha 101**	18	29	29	7	28	29	27	23	7	28	24	20.5	29	28	26	29
**Sakha 102**	26	31	32	9	30	31	15	30	12	32	31	12	30	30	31	30
**Sakha 104**	13	20	18	5	24	20	23	14	5	23	15	4	17	21	18	19
**Sakha 105**	27	25	31	15	32	25	32	29	15	31	29	26.5	22	29	30	24
**Sakha 106**	24	28	30	12	31	28	28	26	11	30	27	12	25	32	32	25
**Sakha 107**	10	15	10	6	22	15	13	10	6	18	11	20.5	15	16	14	16
**Sakha 108**	14	33	28	4	29	33	31	20	3	27	21	12	32	33	25	32
**Sakha Super 300**	11	32	22	2	25	32	30	15	2	25	14	20.5	27	24	21	27
**IRAT 170**	15	22	21	26	18	22	21	12	24	14	12	31.5	24	23	20	18
**A22**	17	7	9	18	17	7	8	13	20	16	17	20.5	4	5	7	5
**Nerica 9**	29	5	19	24	27	5	10	27	25	26	28	4	2	7	10	3.5
**IET 1444**	3	19	7	14	1	19	29	3	13	1	3	20.5	19	11	9	20.5
**Nerica 7**	22	3	8	22	16	3	5	18	21	20	20	12	7	9	8	7.5
**Moroberekan**	32	1	26	25	26	1	1	32	28	29	32	26.5	5	6	11	3.5
**GZ 1368**	5	21	13	16	15	21	26	7	16	5	4	12	20	14	13	15
**Azucena**	21	24	24	31	7	24	25	19	29	10	16	26.5	28	27	27	28
**IRAT 112**	30	12	16	29	21	12	6	28	31	22	26	20.5	14	18	23	14
**N22**	6	26	14	20	12	26	34	9	18	4	6	4	23	20	16	20.5
**IR65600-77**	4	8	5	10	9	8	17	4	8	7	5	12	8	10	6	9
**IR69116**	25	16	17	28	14	16	3	24	27	17	23	29.5	16	17	19	17
**IR12G3213**	8	4	4	8	10	4	7	6	9	12	8	12	3	3	5	2
**IR69432**	19	11	11	21	13	11	2	17	23	15	19	29.5	10.5	12	12	10

**IR6500-127**	28	18	20	32	19	18	16	25	32	19	25	12	21	22	28	23
**IR11L236**	7	2	3	11	6	2	4	5	10	11	7	26.5	1	2	2	1
**IR12G3222**	9	6	6	13	11	6	11	8	14	13	9	34	6	4	3	6
**Sakha 109**	20	14	15	19	23	14	24	21	17	24	22	4	13	15	15	13
**Vandana**	23	27	25	30	5	27	19	22	30	9	18	20.5	31	34	29	31
**Dular**	31	23	27	34	20	23	14	31	34	21	30	31.5	26	26	33	26
**Sakha 103**	34	13	33	33	33	13	22	34	33	33	33	4	12	13	24	12
**Giza 181**	12	17	12	23	8	17	20	11	22	8	10	12	18	19	17	22
**Giza 182**	16	30	23	27	2	30	12	16	26	6	13	20.5	33	25	22	33

Table S6: Favorable and unfavorable classification of the environments

ENV	Y	index	class
E1	946.1507	171.6767	favorable
E2	961.5667	187.0927	favorable
E3	525.907	-248.567	unfavorable
E4	525.5417	-248.932	unfavorable
E5	983.306	208.832	favorable
E6	986.2924	211.8184	favorable
E7	634.0729	-140.401	unfavorable
E8	632.9546	-141.519	unfavorable
